# Chinese University Students’ Perspectives on Help-Seeking and Mental Health Counseling

**DOI:** 10.3390/ijerph19148259

**Published:** 2022-07-06

**Authors:** Xuan Ning, Josephine Pui-Hing Wong, Silang Huang, Yina Fu, Xiaojie Gong, Lizeng Zhang, Carla Hilario, Kenneth Po-Lun Fung, Miao Yu, Maurice Kwong-Lai Poon, Shengli Cheng, Jianguo Gao, Cun-Xian Jia

**Affiliations:** 1 Faculty of Humanities and Social Sciences, Hong Kong Baptist University-Beijing Normal University United International College, Zhuhai 519087, China; xuanning@uic.edu.cn; 2 Daphne Cockwell School of Nursing, Toronto Metropolitan University, Toronto, ON M5B 2K3, Canada; isabella.huang@ryerson.ca; 3 Department of Social Work, Shandong University, Jinan 250100, China; ynfu@sdu.edu.cn (Y.F.); yumiao@sdu.edu.cn (M.Y.); chengsl@sdu.edu.cn (S.C.); gaoj@sdu.edu.cn (J.G.); 4 Department of Sociology, University of Jinan, Jinan 250024, China; sl_gongxj@ujn.edu.cn; 5 Department of Philosophy, Shandong Normal University, Jinan 250014, China; zhanglizeng6666@126.com; 6 School of Nursing, The University of British Columbia, Vancouver, BC V6T 1Z4, Canada; carla.hilario@ubc.ca; 7 Department of Psychiatry, University of Toronto, Toronto, ON M5S 1A1, Canada; ken.fung@uhn.ca; 8 School of Social Work, York University, Toronto, ON M3J 1P3, Canada; mklpoon@yorku.ca; 9 Department of Epidemiology, School of Public Health, Cheeloo College of Medicine, Shandong University, Jinan 250012, China; jiacunxian@sdu.edu.cn

**Keywords:** Chinese university students, help-seeking, mental health, mental health counseling

## Abstract

Psychological distress and mental illness have become increasingly pervasive among Chinese university students. However, many university students who need mental health treatment or psychological support do not actively seek help from professional counselors or service providers, which could lead to poor mental health outcomes. To promote help-seeking, we undertook a qualitative study to understand Chinese university students’ perspectives on help-seeking and mental health counseling. We conducted 13 focus group interviews with students in six universities in Jinan, China, and altogether 91 (62%) female students, and 56 (38%) male students participated in the study. Our results indicate that students’ misconception and distrust of on-campus counseling, stigma of mental illness, low mental health literacy, and hard-to-access mental health services are the major barriers that impede students help-seeking behaviors. Internal struggles and systematic and organizational barriers are identified to shed light on future work to promote mental health literacy among Chinese university students.

## 1. Introduction

Psychological distress and mental illness have become increasingly pervasive among university students [1,2,3]. Existing research indicates that depressive symptoms among Chinese university students have been on the rise [4,5]. Prolonged psychological distress in Chinese university students has been associated with a wide range of negative outcomes such as reduced academic performance, anxiety, depression, and suicidal behavior [6]. The prevalence of mental health problems such as depression and suicide attempts among Chinese universities is alarmingly high in the recent decade. Some studies reported that the prevalence rates of mental illness among Chinese university students were as high as 20% to 30% [2,7,8,9].

However, many university students who need mental health treatment or psychological support do not actively seek help from professional counselors or service providers. Although the Chinese central government has emphasized university students’ mental health education and built infrastructure to provide student counseling services inside higher education institutions [10], most university students do not access on-campus mental health support. In 2018, the Ministry of Education of the People’s Republic of China (MOE) issued its new guidelines on promoting mental health in higher institutions, and regulated that the ratio of on-campus counselors to students should not be lower than 1:4000, and each higher institution should have at least two on-campus counselors [11]. Though many universities hired part-time counselors to meet these requirements, this is still far from what students need. For example, Tsinghua University, China’s very top university, has 38,000 students, but only 11 full-time and 21 part-time counselors work there, which leads to 4027 counseling times per counselor each year [12]. Once students sign up for a counseling service on campus, though it is for free, the waiting time could range from a week to months, depending on the urgency of the case and the turnover of the counselors. Students tend to conceal their mental health problems and seek support from their friends or family rather than professional psychological counseling [10,13]. Studies on university students’ help-seeking behaviors in other countries show similar trends. In general, students who experience mental health challenges access two types of support. First, they seek informal support from close friends or family members, and second, they access formal support from mental health services and on-campus counseling centers [14,15,16].

Although delays in accessing professional support are common worldwide, Chinese university students are comparatively much more delayed in accessing care when they experience mental health problems [17,18,19]. In China, studies indicate that people with mental health problems account for 63% of suicide deaths while only 7% of them have sought professional help before committing suicide [20]. The mental health literacy level among adults across different cities in China remain low to moderate, which results in different populations moderate to high levels stigma against mental illness [21,22]. Evidence shows that 91.8% of individuals diagnosed with mental disorder in China never sought mental health care [23]. How gender is related to stigma still needs more research, although current research shows that compared with males, females tend to be more willing to use mental health services [24]. In the context of higher education, students often try to conceal or solve their mental health challenges by themselves. Professional help is the last resort that university students tap into when they are in psychological crisis [25]. However, research shows that students’ higher levels of depressive symptoms reduce their likelihood of seeking help from parents and friends, as their levels of withdrawal and indifference toward help-seeking tend to increase together with their depression, which makes it increasingly difficult for students to articulate their feelings and reach out for help. Moreover, no evidence supports that an increased level of depression is associated with increased help-seeking from professionals among Chinese university students, which might be explained by the cultural stigma attached to mental health problems [26].

Early identification and intervention for psychological distress are critical to mitigate the negative consequences of untreated mental illnesses [27,28,29,30]. Delayed diagnosis and treatment can put those affected at risk of adverse outcomes such as mental health deterioration, delayed recovery, reduced likelihood of long-term recovery, and increased mortality rate [19,31,32,33]. University students’ mental health is a key concern of decision makers in higher education and society at large in China. Helping university students to develop mental health literacy, recognize their psychological distress, acquire timely assessment and diagnosis, and skills in managing mental illness is urgently needed. For this reason, it is important to identify the factors that influence university students’ reluctance to seek professional mental health services. To gain a better understand of these factors, we conducted 13 focus groups (FG) with 147 students in six Chinese universities. This article focuses on the health-seeking attitude and perspectives of the participants.

## 2. The Present Study

This article reports the results of the qualitative data collected during Phase One of an implementation science project that aims to improve university students’ mental health in China. It is a 5-year research project built on a collaborative partnership between researchers in Canada and China. Six universities and one provincial mental healthcare hospital in Jinan are partners in this project. Linking Hearts consists of three phases. Phase One focused on examining the sociocultural contexts of mental health among university students. We used mixed methods including questionnaires and focus groups to assess the mental health status, health literacy and help-seeking attitudes of university students [34]. The results of Phase One have been used to contextualize and modify the Acceptance and Commitment to Empowerment (ACE) intervention proven to be effective in reducing mental illness stigma and promoting resilience when used with Chinese and other ethno-racial communities in Canada. ACE comprises two components, namely, Acceptance and Commitment Therapy (ACT) and Group Empowerment Psychoeducation (GEP), which aim to reduce people’s stigma against mental illness and increase the collective empowerment to promote mental health. However, ACE was developed and tested among different communities in Canada; thus, it is crucial to first conduct a contextual assessment and analysis of the mental health needs of the students in Jinan to effectively adapt ACE in an intervention that caters for the needs of the university students in Jinan [34]. Phase Two consists of implementing and evaluating the effectiveness of the ACE intervention with university students and mental health professionals. Phase Three focuses on knowledge dissemination.

## 3. Method

### 3.1. Theoretical Framework

In this paper, we integrated two health behavioral change theories to guide our data analysis. First, in the Help-Seeking Model, Rickwood and colleagues [35] postulate that in order for individuals to seek mental health support, they need to become aware that they have a problem and acknowledge that they need help. In addition, they need to know where and how to access resources that address their health needs. Most importantly, they must be willing to disclose personal information in the process of help-seeking. While this model offers specificity of individual decision-making factors, it does not address organizational and structural factors that facilitate or impede help-seeking. Second, we drew on the Theory of Planned Behavior [36], which suggests that individuals are more likely to seek help when they acquire more knowledge about help-seeking, experience changes in subject norms (e.g., reduced stigma of mental illness), receive confirmation from significant others that they need help, and perceive that they are capable of seeking help, i.e., perceived behavioral control. The integrated application of these two behavioral theories enables us to examine the complex factors that are mutually reinforcing in shaping university students’ attitude toward help-seeking.

### 3.2. Recruitment and Data Collection

The study has received ethics approvals from Ryerson University, Shandong University and 10 other partner universities and healthcare organizations. To achieve maximum reach of students, we used both online and offline approaches. For online recruitment, information regarding this project was posted and distributed in WeChat groups formed to disseminate health promotion information to students in different universities. The selection of specific WeChat groups was based on consultation with student leaders. For offline approaches, posters with information about the study were posted at the entrance of canteens and student dormitories for recruitment purpose. Snowball sampling was also used to recruit students through participants who attended the first round of the focus groups. All students interested in taking part in the study were directed to email the project coordinators. The recruitment materials indicated clearly that participation was voluntary and based on students’ willingness. Participants were informed that confidentiality, anonymous coding of their names, and the choice of immediate withdrawal were guaranteed. Each of the six universities set the goal of recruiting 24 participants. The inclusion criteria were that participants should be above 18 years old and studying in one of these six partner universities in Jinan. In total, 147 students were recruited and took part in 13 focus groups. There were 91 (62%) female students, and 56 (38%) male students.

Written consent was obtained from each participant prior to the focus groups. Interviews were conducted in the participants’ first language, i.e., Mandarin. Each focus group lasted about two hours on average. Each focus group was conducted by two to three facilitators, who were research team members or graduate trainees not known to the participants. An interview guide was used by focus group facilitators to explore participants’ conceptualization of mental health and mental illness, their perceptions of students’ mental health issues and needs, their perspectives on programs and services useful to students, and their suggestions for this research project. All interviews were audiotaped and transcribed verbatim in Chinese for data analysis. The transcribed interviews were cross-checked by team members to ensure accuracy. Then, the Chinese transcripts were translated into English by the first author and reviewed by the second author, who are bilingual and have been immersed in the research processes of this project. In addition, the first author has lived experiences in Jinan as a local, received bilingual Chinese-English undergraduate and graduate education in China, and living in Canada during Phase One of the study. Her lived experiences and expertise enabled her to work with the team to translate and interpret the contents beyond linguistic equivalency to integrate cultural translation based on the contexts relevant to university students in Jinan [37].

### 3.3. Data Analysis

Data analysis was guided by McCracken’s [38] thematic coding with NVivo. Transcripts were independently reviewed in details by the first two authors to gain familiarity with the participants’ narratives. Then, they undertook a line-by-line open coding of key ideas expressed by the participants as well as concepts based on the research questions. The open codes were then organized into themes based on their similarity and distinctiveness. Team members then met to discuss, compare, and interpret the finalized themes. This two-step data analysis process enabled us to draw on the analytical interpretation of different team members to arrive at the best explanation for participants’ narratives. This process helped us gain deeper insights into the issues and enhanced the trustworthiness of the results [39]. Thematic analysis enabled us to provide a full description of participants’ accounts to enhance the contextual transferability of this study [40].

## 4. Results

The results of this study show that university students encounter a number of barriers that deter them from seeking professional mental health services. The barriers included a lack of trust in counselors, stigma towards mental illness, low mental health literacy, and inaccessibility to mental health services. Among these barriers, a lack of trust in counselors and stigma towards mental illness are the leading factors that account for students’ unwillingness to seek professional help.

### 4.1. Misconception and Distrust of On-Campus Counseling

In sharing their perceptions and perspectives about accessing counseling, participants expressed three key concerns about their hesitation to seek help, and their perceptions were quite complex.

Many participants felt that counselors, especially on-campus counselors, did not have the professional expertise required to provide the support required to meet their needs. They explained that many on-campus counselors were academic staff whose main roles were teaching and doing research, and not counseling students. However, due to the disproportionately high number of students needing mental health care and low number of on-campus counselors, academic staff were expected to function as on-campus counselors even though their counseling experiences were limited.

“I have contacted many on-campus counselors who are academic staff… I feel that their expertise in counseling has not reached a high professional standard. Thus, they would ask direct questions like, ‘What do you feel you wish to do next? What do you think you should do as your next step?’ These questions made me feel like I was being interrogated, which made me uneasy.” (University #5, FG #2, student #4, female).

The majority of participants resonated with this student, who did not consider counseling as an effective therapeutic modality. They believed that their mental health challenges could not be resolved through “talking”. Only 14 out of the 147 participants from the six universities perceived counseling to be useful only for people with mental illness like depression, which could be diagnosed and treated in a standardized and systematic manner. They did not believe that counseling is effective in addressing stressors associated issues specific to personal situations and contexts. Furthermore, less than one tenth of students felt that effective counseling requires the counselors to have similar lived experiences to truly understand what university students were going through. A couple of participants suggested that counselors often stayed on their intellectual high horse, using an overly general professional stance, which seldom led to “person-centered” practical solutions.

“Some students compare psychologists and counselors to ornaments or decorations, and they consider what the counselors say in counseling as flowery language or rhetoric. They do not trust psychologists. They feel that psychologists do not have the professional expertise as physicians or surgeons, and therefore are not trustworthy. They do not believe psychologists can really help them with their mental health problems.” (University #6, FG #1, student #9, male).

In addition to the lack of confidence in the counseling profession, participants also identified worries regarding a potential breach of confidentiality and privacy as a source of distrust in counseling. Their key concerns included the requirement to provide personal information during registration; the risk of being seen by others when accessing mental health counseling; and concerns that counselors might not keep what was shared during counseling confidential. The general procedure for a university student to go through in order to sign up for on-campus counseling service is as follows. First, this student needs to fill in a survey that require their personal information including name, contact information, mental illness history, and purpose of seeing a counselor. Then, this survey is sent to a counselor for triage, and the counselor who decides to follow up on this case will contact the student. Students are told that their personal information and the conversation with the counselors will be kept confidential before the counseling session. However, there are few guidelines to inform counselors on the breach of confidentiality in terms of treating mental illness for adolescents in China. Chinese counselors reported that they considered it to be ethical to breach confidentiality if they found situations like daily drug use or suicidality [41,42].

“You need to register with your real personal information when you look for on-campus counseling service, which means your privacy might not be maintained, and students are concerned about losing their privacy.” (University #4, FG #2, student #3, female).

Some participants considered on-campus counseling particularly “risky” because the counselors might know their peers, professors and other personnel at their university. As anonymity and privacy could not be totally ensured, students were unwilling to seek help from on-campus counselors. One participant, who resonated with the discussion on privacy, shared that her step-mother abused her and her brother. Although the experience was painful and stressful, she did not talk to any counselors as she worried that her situation would become known to people outside of her family.

“I have mentioned this to strangers. Like, if I happen to be chatting to a fellow passenger on the bus, I may mention my problems because I feel that I would never run into them again in the future… Even if they share with others, they do not know my name or real information about me, so the level of confidentiality is higher than talking to on-campus counselors. I need this level of confidentiality. I don’t want my peers to know about my family situation because they may view me differently and treat me differently.” (University #2, FG #2, student #9, female).

Difficulty in building rapport with on-campus counselors was also identified as another reason that many students were not willing to seek counseling service when they were in need. In many Chinese universities, student advisors (*fu-dao-yuan*) also function as on-campus counselors. Supposedly, student advisors are familiar with all aspects of student life on campus. They are the first go-to person for all matters that students are concerned about. However, there seemed to be a lot of distrust toward the student advisors due to the power they hold over the students.

“I think lots of students do not like their student advisors very much, for instance, students from my major maintain a very negative attitude toward our student advisor.” (University #2, FG #1, student #3, female).

However, not all participants held negative perceptions about on-campus counseling. Some of them recognized the potential role of counseling in students’ mental health.

“Talking to friends about their problems would comfort students to some extent but would not help with problem-solving. And I think seeking help from professionals could really help students solve their problems.” (University #2, FG #1, student #6, male).

It also appeared that participants who were open to learning more about their own mental wellbeing, had a more neutral perspective of on-campus counselors.

“For me, my curiosity about my own mental health would motivate me to talk to an on-campus counsellor, so that I would know my mental health status better. Also, If I do want to solve my problems, I will seek help from on-campus counselling.” (University #2, FG #2, student #3, female).

The participants’ responses indicated that their attitude toward on-campus counseling was quite diverse and complex.

### 4.2. Stigma of Psychological Counseling

In addition to misconceptions about psychological counseling, stigma of mental illness was also a key barrier for students to seek counseling services. Many participants had the perception that only people with “severe” mental illness would need to see a counsellor. Some even worried that one’s mental health could worsen after seeing a counsellor.

“Maybe initially it was only a small problem that could be easily resolved by confiding in and chatting with a close friend. When you go and seek help from a counsellor, your problem may become magnified, and you may end up experiencing some unnecessary negative effects.” (University #2, FG #2, student #7, female).

For some participants, needing mental health support and seeking counseling were associated with the stigma of personal weakness.

“I think that we all feel ashamed of seeking help from a counselor or psychiatrist. It makes us wonder if we have serious psychological problems. It also makes us doubt our own ability to manage stress, or question if we do have inferior mental health quality.” (University #6, FG #1, student #7, male).

Other participants expressed fear of being stigmatized or discriminated by others. One participant indicated that he would never go to his student advisor for counseling even if he were faced with severe mental health challenges.

“I would rather keep my mental health challenges all to myself rather than talk to my student advisor or thesis supervisor. What if they regard me as weird and abnormal and treat me differently? That would bring a huge shame on me, and I do not want to lose face for this humiliation. Besides, if they find out there is something wrong with my mental health, they would not let me participate in my thesis defense and they would not let me graduate on time.” (University #1, FG #1, student #11, male).

For many participants, stigma could lead to real, negative impacts on their life because student advisors hold a lot of decision-making power. They can determine many aspects of students’ life on campus, including their eligibility to join the Chinese Communist Party, obtain honors and awards, and participate in school activities.

Other participants suggested that the pressure to present themselves as mentally healthy and problem-free came from other students because stigma and rejection can affect all aspects of their life on campus, as one participant explained:

“Most people do not think that seeking counseling service is a normal thing to do. Most of the time, when someone has gone for counseling service, they would say, ‘You went to see a counselor. Are you sick?’” (University #1, FG #2, student #4, male).

The implied rule of ‘normalcy’ based on peer judgement, reinforced stigma toward mental illness, which creates barriers to help-seeking among students.

However, peer influence could also open up space to challenge stigma and discrimination.

“Some of my good friends have tried on-campus counselling before. They found on-campus counselling very useful, not only in helping them with the specific mental health challenge they were experiencing, but also other issues in their everyday life. My friends found the counseling experience very inspiring to them, so they highly recommended me to use on-campus counselling if I were to encounter any mental health challenge.” (University #1, FG #1, student #5, female).

It appeared that when students shared their positive counseling experiences with their peers, they were able to reduce stigma toward mental illness and help-seeking.

### 4.3. Low Mental Health Literacy

Despite concerns about stigma and discrimination, some participants recognized that their perceptions and attitudes toward counselling might be related to their understanding of mental health.

“I think it would be easier for students who have certain level of mental health literacy to seek help from on-campus counsellors or professional mental healthcare services when they encounter challenges. For example, students who major in psychology or social work are familiar with counselling service, and because of their good understanding and knowledge of mental illness, they barely attach any stigma to counselling service; instead, they regard seeking help from counsellors as quite a normal thing to do.” (University #2, FG #2, student #4, female).

However, many participants felt that existing efforts by university administration to disseminate mental health information and related services were inadequate. They felt that students could benefit from learning more about mental health.

“We have very few opportunities to learn about mental health in our everyday life on campus. This gives us an impression that mental health is not relevant to us. When we encounter psychological challenges, we may not recognize that these challenges are related to mental health. Even if we want to seek counseling, we know so little about counseling resources on campus that it takes a lot of efforts to figure out about when, where, how, and who can really help us. Many students are discouraged by this inconvenience and thus do not bother to seek on-campus counseling.” (University #5, FG #2, student #11, female).

The need for more knowledge about on-campus mental health resources was echoed by many participants. One participant cited the example that she and her roommates did not really know where the on-campus counseling office was located. Others indicated that access to mental health resources could be particularly important for first year undergraduate students, who got so excited about everything that they might not even be aware if they have mental health challenges and need help.

“This is particularly true for first year students, who are fascinated with their new life and can find excitement in anything they do, unlike second-or-third year students who have begun to experience many challenges related to their college life and recognize the need for support.” (University #2, FG #2, student #1, female).

Other participants identified the importance of promoting mental health literacy among students as one way to promote help-seeking for mental health care.

“We are influenced by other people’s perspectives. If we are surrounded by people with some foundational mental health literacy, we would consider seeing a counselor as something normal. We would not carry any psychological burden when we access counseling services. However, when everyone around us considers counseling as awful or abnormal, then it would be difficult for us to overcome these hurdles to seek mental health counseling.” (University #1, FG #2, student #3, female).

Some participant pointed out that increasing health literacy would enable students to recognize symptoms of mental health challenges, or the urgency to seek help to address these challenges.

### 4.4. Hard-To-Access Mental Health Services

For participants who wished to seek support to address their mental health challenges, access to psychological counseling was limited. Many participants shared that making an on-campus counseling appointment was too complicated, especially for students who felt they needed urgent support. Usually, students needed to wait at least two weeks till they could meet with the counselors.

“I want to share the obstacles students encounter during their process of counseling-seeking. The truth is that more on-campus counselors are urgently needed. If you try to book an appointment via phone, staff at the counseling center will arrange a meeting for you after two weeks, so they cannot really help you with your urgent need, but your issue might be resolved long before you meet the counselors.” (University #3, FG #2, student #6, female).

In addition, some participants were discouraged by the cumbersome processes of registration, long questionnaire in-person or by phone in primary screening, and waiting in line to meet with the counselors but then the session only lasted for a few minutes. Other participants indicated that the existing number of on-campus counselors was insufficient to meet the huge need for counseling services among students. Therefore, it was difficult to secure a timely appointment with a counselor to address their urgent needs.

“I think the current on-campus counseling is still challenged by disproportionate ratio of few counselors to many students, so that students’ needs cannot be met, which leads to unsatisfactory results.” (University #5, FG #2, student #3, female).

The lack of timely access to on-campus counseling service might help to explain why students turned to their peers for support. Furthermore, the lack of understanding about the time required to go through counseling also created challenges for some students.

“For psychological counseling, we often miss the appointments because of scheduling conflicts, or not paying enough attention to them. And if you only attend counseling once or twice, it doesn’t really have a sustainable effect.” (University #5, FG #1, student #1, female).

When students held high expectations on the outcomes of their first visit to a counselor, they often felt frustrated when they learned that they needed to return to attend more counseling sessions. Some also felt discouraged when they perceived counseling as a time-consuming activity amidst their school and relationship demands.

## 5. Discussion

The results of this study show that university students’ attitudes toward help-seeking and mental health counseling were shaped by complex factors that are interconnected and mutually reinforcing. According to Rickwood and colleagues [35], help-seeking occurs when students become aware that they have a mental health problem and need help. This self-awareness is determined by students’ mental health literacy. In this study, participants shared that most students had limited knowledge about mental health, making it difficult for them to recognize symptoms of mental illness or the need for professional help. In addition, similar to the results of previous surveys of university students [43], many participants reported that they did not know where they could access mental health counseling on campus.

The lack of access to adequate student-centered mental health education also gave rise to other barriers. Low mental health literacy contributed to misconceptions that psychological counseling was only for people with serious mental illness, and that help-seeking was an indicator of personal weakness because ‘normal’ people should be able to deal with their own ‘personal’ problems. These misconceptions created a social expectation of normalcy that could be enacted or internalized as stigma of mental illness, which further discouraged help-seeking among students. The fear of being viewed as abnormal, rejection by their peers, or discrimination by others on campus made it difficult for students to access care even if they were aware of their needs [25,44]. Furthermore, within the context of Chinese culture, stigma is a complex challenge entangled with the phenomena of ‘face-saving’ and ‘face-losing’.

The notion of face, a well-studied construct, refers to “a positive social impression or image that individuals want to claim, maintain, or enhance in the presence of others” [45], (p. 120). Face-saving is achieved and maintained when individuals are able to fulfill their expected social roles and responsibilities [46,47]. The prioritized social expectation on university students is academic success. Despite having to deal with many stressors, such as living away from home, establishing new interpersonal relationships, adopting new ways of learning, and worrying about their future, many students are reluctant to seek mental health counseling. Help-seeking is associated with the threat of face-losing, which may result in rejection and discrimination from one’s social networks, as well as self-shame and associated shame experienced by one’s family [48]. Thus, for Chinese university students, the perceived self-efficacy needed for help-seeking is often compromised by the intersecting impact of stigma and face-losing [49]. This finding suggests the need for a new area of research that critically examines how structural inequality and social discrimination shape the collective conceptualization of ‘face’ as a cultural phenomenon.

Aside from the internal struggles experienced by university students, organizational and systemic barriers also impede help-seeking. Echoing previous studies [50,51], the results of this study show that students preferred to talk to their friends over seeking help from on-campus counselors for multiple reasons. First, students worried that confidentiality of their personal information and mental health problems would not be maintained, leading to the consequences of stigma, face-losing, and discrimination that would compromise their academic success and social relationships. Second, students expressed serious doubt toward the competence of on-campus counselors, which is understandable. Due to a shortage of professional mental health counselors, many student advisors (*fu*-*dao*-*yuan*) had to provide psychological counseling, in addition to their roles in managing student affairs, alumni affairs, patriotic education, and moral education [52]. Most student advisors had limited training in mental health and psychological counseling. Furthermore, the close working relationships and power differential between the student advisors and students produced additional worries that impede help-seeking among students. These worries were again associated with the intertwined phenomena of stigma and face-losing, which could negatively impact on the students’ social life and academic achievements. Third, to access on-campus counseling, students were required to provide lengthy personal information, to line up in a space visible to others, talk with a counselor without any established rapport, and needed multiple counselling sessions. These processes also contributed to the anxiety associated with compromised social status and their perceptions that time could not be wasted on talk when they were supposed to focus on academic success.

## 6. Implications and Limitations

Our study results show that on-campus mental health promotion is needed to increase mental health literacy, reduce stigma, and re-define ‘face’ based on the collective values of contribution, compassion and acceptance rather than the normative expectations that further impose stress, shame and fear among students. There is also a need to expand existing mental health resources. At the university level, professional training and consolidation of skills are needed to support effective on-campus counseling. Since students tend to talk to their friends about their problems before reaching out to professional counselors, the development of structured peer-to-peer support and peer counseling programs can contribute to meeting the mental health needs of students. New mechanisms such as online registration, e-booking of appointments, online or telephone counseling, and private counseling spaces may facilitate help-seeking. At the systems level, interprofessional collaboration among student advisors, psychological counselors, mental health nurses, psychiatrists, and other relevant professionals can contribute to expanding professional capacity in mental health care. The mental health of university staff and students will affect one another, and more attention should be paid to university staff’s mental health or else their poor mental health will inevitably have a negative impact on students’ mental health. As young people, including university students, are valuable resources in society, promoting mental health literacy and timely help-seeking among young people is a worthy investment.

Despite its important contributions, this study has some limitations. The study engaged only 147 students from six universities. Furthermore, the study took place in Jinan, a tier-two city in China. Thus, the perspectives of the students cannot be generalized to represent those of all Chinese university students in China, especially those in cities of different tiers. However, the insights gained may be used to inform mental health care among students in similar sociocultural and environmental contexts. Future studies may consider comparing student perspectives across cities of different tiers.

## 7. Conclusions

This is one of the few studies that uses a qualitative approach to explore Chinese university students’ perspectives on help-seeking and mental health counselling. Our research found a lack of trust in counselors, stigma towards mental illness, low mental health literacy, and inaccessibility to mental health services were four major barriers that impeded students from seeking timely help from professional mental health services. University students in China are faced with tremendous pressure to achieve academic success and the demands of establishing and maintaining positive social relationships on campus. Seeking timely mental health support is critical to students’ wellbeing. Through the use of a qualitative approach, we were able to shed light on the contextual and interconnected factors that impede help-seeking among students. Our study results show that on-campus mental health promotion is needed to increase mental health literacy, reduce stigma, and re-define ‘face’ based on the collective values of contribution, compassion and acceptance rather than the normative expectations that further impose stress, shame and fear among students. Future research using qualitative approaches is needed to further explore the strategies required to promote timely help-seeking among university students.
